# Effect of D-ribose supplementation on delayed onset muscle soreness induced by plyometric exercise in college students

**DOI:** 10.1186/s12970-020-00371-8

**Published:** 2020-08-10

**Authors:** Wei Cao, Junqiang Qiu, Tianwei Cai, Longyan Yi, Dan Benardot, Menghui Zou

**Affiliations:** 1 Department of Exercise Biochemistry, Exercise Science School, Beijing Sport University, No. 48 Xinxi Road, Haidian District, Beijing, China; 2 Department of Nutrition, Georgia State University, Atlanta, GA USA; 3 Center for the Study of Human Health, Emory University, Atlanta, GA USA; 4 China Athletics College, Beijing Sport University, Beijing, BJ China

**Keywords:** Muscle soreness, Isokinetic muscle strength, Muscle damage, Oxidative stress

## Abstract

**Objective:**

Previous investigations suggest that appropriate nutritional interventions may reduce delayed onset muscle soreness (DOMS). This study examined the effect of D-ribose supplementation on DOMS induced by plyometric exercise.

**Methods:**

For the purpose of inducing DOMS, 21 untrained male college students performed a lower-limb plyometric exercise session that involved 7 sets of 20 consecutive frog hops with 90-s of rest between each set. Muscle soreness was measured with a visual analogue scale 1-h before, 24-h after, and 48-h after exercise. Subjects were then randomly placed into the D-ribose group (DRIB, *n* = 11) and the placebo group (PLAC, *n* = 10) to assure equivalent BMI and muscle soreness. After a 14-d washout/recovery period, subjects performed the same exercise session, with DRIB ingesting a 200 ml solution containing 15 g D-ribose 1-h before, 1-h, 12-h, 24-h, and 36-h after exercise, and PLAC ingesting a calorically equivalent placebo of the same volume and taste containing sorbitol and β-cyclodextrin. Muscle soreness and isokinetic muscle strength were measured, and venous blood was assessed for markers of muscle damage and oxidative stress 1-h before, 24-h and 48-h after exercise.

**Results:**

In DRIB, muscle soreness after 24-h and 48-h in the second exercise session were significantly lower (*p* < 0.01) than was experienced in the first exercise session. In the second exercise, blood-related markers of muscle soreness, including creatine kinase, lactate dehydrogenase (LDH), myoglobin and malondialdehyde (MDA) in DRIB after 24-h were lower in DRIB after 24-h than in PLAC (MDA, *p* < 0.05; rest outcomes, *p* < 0.01). In addition, LDH and MDA in DRIB were significantly lower (*p* < 0.01) after 24-h in DRIB than in PLAC. No difference was found in isokinetic muscle strength and oxidative stress markers, including superoxide dismutase and total antioxidant capacity, between DRIB and PLAC after 24-h and 48-h.

**Conclusion:**

D-ribose supplementation reduces muscle soreness, improves recovery of muscle damage, and inhibits the formation of lipid peroxides. Young adult males performing plyometric exercise are likely to realize a DOMS reduction through consumption of D-ribose in 15 g/doses both before (1-h) and after (1-h, 12-h, 24-h, 36-h) exercise. These results suggest that appropriately timed consumption of D-ribose may induce a similar alleviation of exercise-induced DOMS in the general public.

## Introduction

Delayed onset muscle soreness (DOMS) is described as ultrastructural muscle damage that occurs following exercise, and is characterized by localized muscular tenderness and soreness [1]. The soreness normally increases in intensity in the first 24-h following a bout of exercise, peaks from 24-h to 72-h post-exercise, then subsides 5 to 7 days post-exercise [2]. The occurrence of DOMS may be caused by a variety of complex morphological factors [3], including: Overexertion of muscles leads to mechanical damage and inflammation [4–6]; neutrophils and macrophages accumulate in the inflamed region, which leads to secondary inflammation and the initiation of the repair process [7, 8]; the inflammatory response increases the concentration of pain-causing substances that include bradykinin, leukotrienes and prostaglandin [9]; mitochondrial respiration during exercise enhances oxidative stress [10]. Intensity exercise increases energy requirement, and causes an increase in inosine monophosphate and in hypoxanthine [11]. The hypoxanthine is then converted to uric acid and oxygen radicals, leading to an increase in reactive oxygen species (ROS) [12]. The increased level of ROS leads to an increased inflammatory cascade [13] that is directly associated with damage to the cell membrane [14]. Therefore, the general characteristics of DOMS includes elevated muscle soreness, decreased muscle function, and lower strength. The internal metabolic characteristics of affected cells include the leakage of skeletal muscle enzymes and proteins and an increased level of oxidative stress that is typified with increased levels of serum creatine kinase (CK), lactate dehydrogenase (LDH), and myoglobin (MB). In addition, large amounts of ROS are produced, and the malondialdehyde (MDA) level is elevated as a marker of lipid peroxidation [15–19].

Because DOMS is associated with an extended post-exercise recovery time, it is important to develop strategies that enable an enhanced muscular capacity for restoration of adenosine triphosphate (ATP) and functionality. It has been hypothesized that the pentose phosphate pathway (PPP) and purine nucleotide pathway may play a key role in DOMS recovery [20]. D-ribose is the intermediate product of PPP in glucose metabolism, and it has been suggested that D-ribose ingestion accelerates the formation of phosporibosylpyrophosphate and increases the rate of adenine nucleotides synthesis, thus accelerating the de novo and salvage synthesis of ATP [21, 22]. The enhanced recovery rate of muscle ATP could then shorten the DOMS-associated recovery period following high intensity exercise by enhancing the recovery of the muscle cell membrane and reducing ROS-associated stress. No prior controlled studies have been conducted on the effects of D-ribose supplementation on exercise-induced DOMS. Nevertheless, the potential benefits of D-ribose supplementation for improving exercise performance and/or recovery [22–24], and for reducing markers of ROS production [25] has been explored, though some studies didn’t support this hypothesis [24, 26–28]. Seifert et al. [24] suggested that a failure to support the potential benefit of D-ribose may be due to the difference of fitness level of subjects. Untrained individuals appear to have no ability to recover sufficiently on subsequent days after acute, repeated bouts of exercise. Therefore, our research project selected untrained individuals to better understand the effect of D-ribose on DOMS.

D-ribose is classified as a “generally considered safe” substance, making it a safe additive for human [29] and animal [30] consumption. It has been suggested that the maximum safe oral dose of D-ribose is 200 mg/kg/h [22, 31], with more than 200 mg/kg/h of D-ribose elevating diarrhea risk [28, 31].

DOMS is most likely to be caused by eccentric muscle contractions and/or unfamiliar forms of exercise [2, 3]. In general, eccentric exercise (e.g., walking down the stairs or down hills) is more likely to cause muscle damage and DOMS than concentric exercise (e.g., walking up the stairs or up hills), particularly if the eccentric exercise is performed at high intensity [3]. Plyometric exercise, which represents a rapid deceleration of the body followed by a short transitional phase that is followed by acceleration in the opposite direction [12, 32], involves eccentric contraction of muscles and may result in high risk of DOMS [33, 34]. As a plyometric movement pattern similar to squat jump, the frog hop is a common exercise for both athletes and minimally trained individuals, and is widely used to develop lower-limb strength in China.

D-ribose has been used by Chinese athletes to improve performance. This is the first study to assess the effect of D-ribose supplementation on exercise-induced DOMS. To pursue this area of investigation, we determined and measured the primary biomarkers reflecting muscle performance, muscle damage, and oxidative stress that are related to DOMS, and assessed the effect of D-ribose supplementation on these DOMS-related factors.

## Methods

### Subjects

Twenty-one untrained healthy male college students volunteered as subjects for this study. The inclusion criteria included: 1) in good health, with no chronic diseases such as hypertension, diabetes, cardiovascular disease, or elevated cerebrovascular risk; no lower limb injury; no other diagnosed diseases; 2) no regular and organized exercise training over the past 6 months; 3) no more than 2 exercise sessions per week for 30-min in the past 6 months; 4) no exercise-relevant nutrient supplements were consumed within 1 month of the study initiation; 5) no regular participation in massage or water bath to relieve fatigue and/or enhance relaxation. The study protocol was approved by the Internal Review Board of Beijing Sport University (BSU IRB). All subjects were informed of the aims of the study and were asked to sign an informed consent.

### Study design

Participating subjects were asked to complete the Physical Activity Readiness Questionnaire and were measured for height and weight. They were then taken to the indoor track for the first plyometric exercise session that aimed to purposefully induce DOMS in the lower limbs. Since muscle soreness is a characteristic of DOMS, subjects were matched according to BMI and muscle soreness after exercise, and were randomly assigned to either the D-ribose (DRIB, *n* = 11) or the placebo (PLAC, *n* = 10) groups to assure group equivalence. No significant differences in BMI and muscle soreness were found between groups at baseline (Table 1). A single-blind protocol was used in this study, with subjects unaware if they were assigned to the experimental or control group.

**Table 1:** Subjects’ characteristics for two groups^a^

Group	DRIB	PLAC	Group *p*-value
N	11	10	
Age (yr.)	21(2)	21(2)	0.448
Height (cm)	175.36(5.20)	177.30(2.06)	0.274
Weight (kg)	72.6(7.1)	70.9(9.4)	0.637
BMI (kg/m^2^)	23.6(2.2)	22.5(2.8)	0.327
Muscle soreness			
Baseline	1.5(0.7)	1.8(0.4)	0.187
P 24	5.4(1.6)	6.0(1.9)	0.417
P 48	5.3(1.6)	5.3(2.5)	0.977

*DRIB* D-ribose group, *PLAC* Placebo group

^a^There was no significant difference between the two groups on these variables

**Fig. 1:**
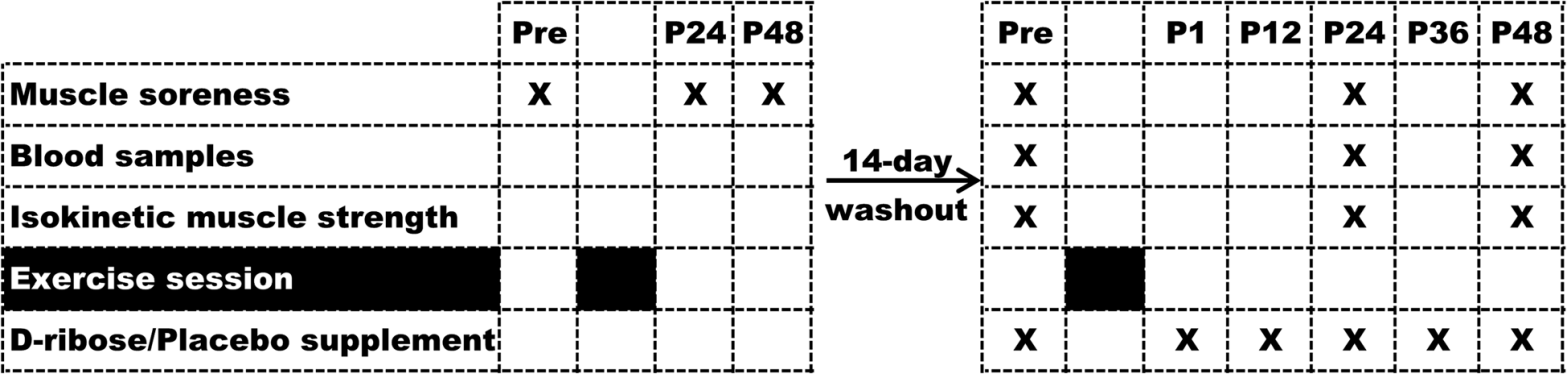
Experimental design

Two weeks after the initial evaluations, all subjects were taken to the indoor track again for the same plyometric exercise session as in the first round. DRIB was supplemented with D-ribose 1-h before, 1-h, 12-h, 24-h and 36-h after exercise; PLAC was supplemented with the placebo (sorbitol, β-cyclodextrin), which was provided at the same times as DRIB. Measurement of muscle soreness, isokinetic muscle strength and venous blood-related markers were obtained 1-h before, 24-h and 48-h after exercise. Subjects were instructed to avoid vigorous exercise within 1-week before the study and to avoid consuming carbonated drinks, alcohol, caffeine and other substances that may affect the results within 2-h before the tests. Subjects were asked to avoid other training and nutritional supplementation during the study. Fig. 1 provides a summary of the experimental design.

### DOMS induction protocol

**Fig. 2:**
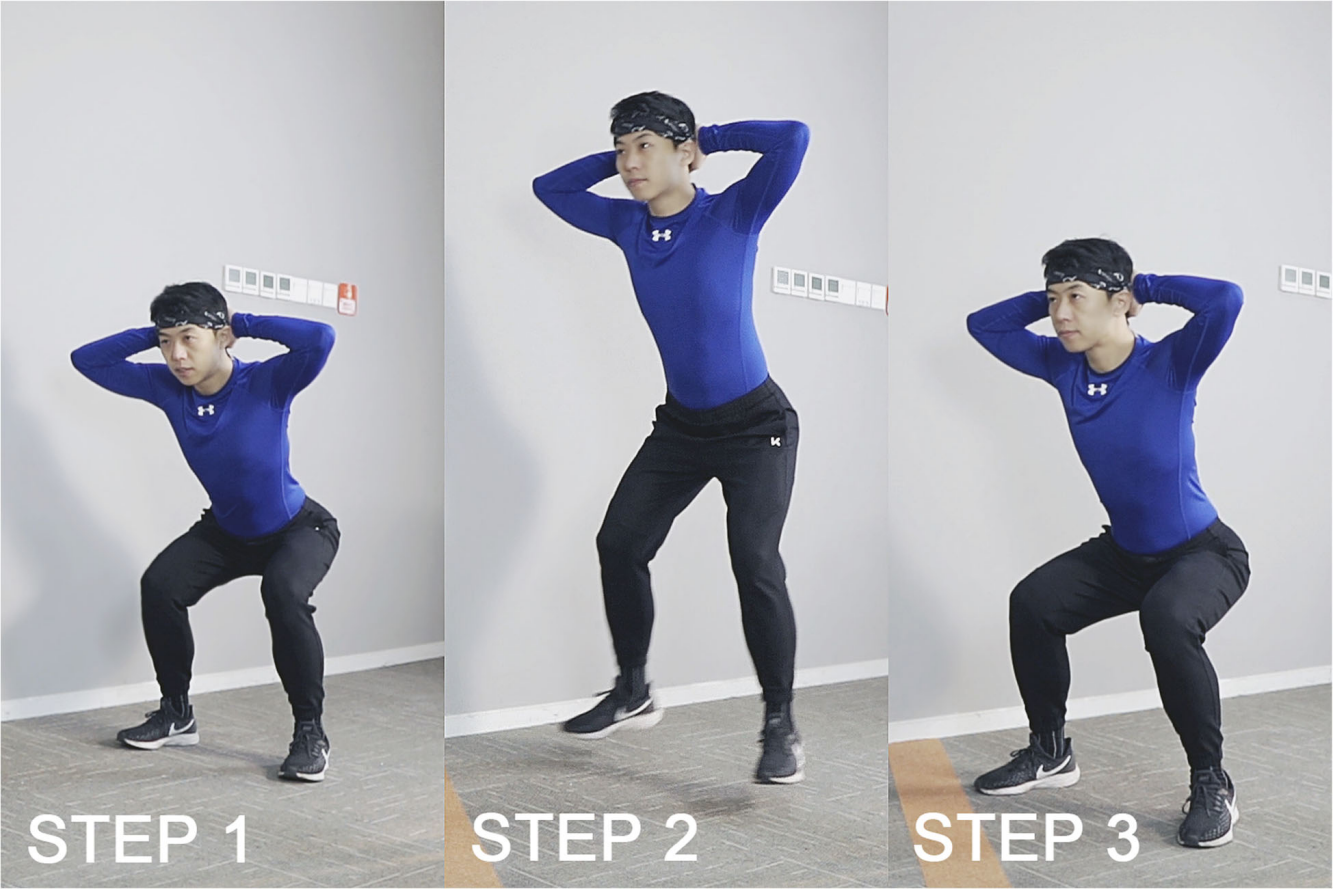
The action essentials of the frog hop. STEP 1: Stand with two hands behind the head and feet shoulder-width apart, squat down, keep the torso upright and the head and chest up. STEP 2: Jump forward, avoid unnecessary high jump. STEP 3: When the feet touch the ground, use the legs to cushion the impact of landing.

Subjects were taken to the indoor track, performed a 5-min warmup jog, rested for a moment and then performed the DOMS initiating exercise. The plyometric exercise protocol used in this study to induce DOMS in the lower limbs consisted of 7 sets of 20 consecutive frog hops, with 90-s of rest between each set. The action essentials of the frog hop include: The starting position involves standing with two hands behind the head and feet shoulder-width apart, squat down, keep the torso upright and the head and chest up. Once in this position, subjects were asked to jump forward, avoiding unnecessary high jump. When the feet touch the ground, the legs are used to cushion the impact of landing (Fig. 2). A total distance of 30-m was required for each set. During the interval, subjects walked slowly back to the starting line and were not allowed to stretch. All subjects were monitored under the supervision of two experimenters. Anyone who could not continue to exercise after being encouraged was classified as fatigued and his exercise was terminated. All subjects completed two full plyometric exercise sessions according to instructions.

### Muscle soreness

Subject muscle soreness was assessed by the same experimenter 1-h before, 24-h and 48-h after exercise using the Visual Analogue Scale (VAS), which is a Likert-based scale identifying the severity of soreness [35–37]. The scale has a total of 10 points, with ‘0’ indicating no soreness and ‘10’ indicating extreme soreness. To familiarize with the VAS scale, subjects were provided with an unmarked VAS prior to evaluation. Then they walked a distance at a regular speed. Before they stopped walking, subjects indicated the point on the scale that corresponded to the soreness they felt while walking.

### Isokinetic muscle strength

The test was measured 1-h before exercise, and again 24-h and 48-h after two plyometric exercise sessions. The knee flexor-extensor muscles of the dominant leg were measured at an angular velocity 60°/s with an IsoMed 2000 dynamometer (D&R Ferstl GmbH, Hemau, Germany). All measures were obtained by the same researcher and were in accordance with the operation manual [38]. To assess range of motion, subjects sat on a seat with 75° hip flexion, with range of motion set to reach from 10 to 90°. The knee articulation axis was the same as the dynamometer mechanic arm lever axis. A Velcro strap was fixed to the distal portion of the tibia and the length of the dynamometer arm varied according to the length of the subject’s leg. After the subject was fixed on the training seat, the weight of the tested leg in a relaxed state at terminal extension was measured and a gravity correction was made. Subjects exercised 5 times with submaximal intensity to familiarize themselves with the test process. Then they were asked to perform 5 reciprocal maximum flexion extension repetitions. Strong verbal encouragement and visual online feedback were provided during testing to assure maximum effort. Flexor peak torque (FPT), extensor peak torque (EPT), flexor total work (FTW) and extensor total work (ETW) were recorded.

### Supplement intervention

At the 5 time points of 1-h before, 1-h, 12-h, 24-h, 36-h after exercise, DRIB was supplied with 15 g of D-ribose (Cheng Zhi Life Science Co., Ltd., Beijing, China) and PLAC with supplied with the same dose and taste of a calorically equivalent sorbitol and β-cyclodextrin containing beverage. Supplements were powders, which were mixed in 200 ml of drinking water for oral administration.

### Blood analyses

Blood samples (5 ml) were obtain from the ulnar vein 1-h before, 24-h and 48-h after exercise. The samples were collected in gel serum tubes (Vacuette, Frickenhausen, Germany) and centrifuged at 3500 rpm in an Eppendorf Centrifuge 5417R (Eppendorf AG, Hamburg, Germany) for 10-min at 4 °C to obtain serum, which was stored at − 80 °C for future analysis.

Blood samples were analyzed for creatine kinase (CK), lactate dehydrogenase (LDH), myoglobin (MB), superoxide dismutase (SOD), total antioxidant capacity (T-AOC) and malondialdehyde (MDA). CK, LDH, SOD, T-AOC and MDA were assessed in duplicate using commercially enzymatic kinetic kits (Nanjing Jiancheng Bioengineering Institute, Jiangsu, China) with a visible 721 spectrophotometer (Shanghai optical instrument factory, Shanghai, China). Coefficients of variation were 1.5, 1.5, 5.1, 3.6, 2.3%, respectively. MB was determined in duplicate using commercially available ELISA kits (Nanjing Jiancheng Bioengineering Institute, Jiangsu, China). Coefficients of variation was 9.9%. Each completed assessment was performed according to manufacturer instructions.

### Statistical analyses

Data were analyzed using SPSS for windows, version 19.0 (SPSS Inc. Chicago, IL, USA), and presented as means and standard deviations. One-way ANOVA was performed on VAS score between exercise sessions. Two-way repeated measures ANOVA was performed on all markers before, 24 h and 48 h after exercise between groups and within groups. Data were analyzed using absolute changes from before exercise (pre) ± 95% confidence intervals (CIs); If 95% CI didn’t cross 0 (i.e., 0 change), it was considered significant.

## Results

All subjects completed the two plyometric exercise sessions and tests. At the first test, there were no significant differences between groups in the markers of muscle soreness (Table 1), isokinetic muscle strength, muscle damage, and oxidative stress (data not shown) (*p* > 0.05).

### Muscle soreness

**Fig. 3:**
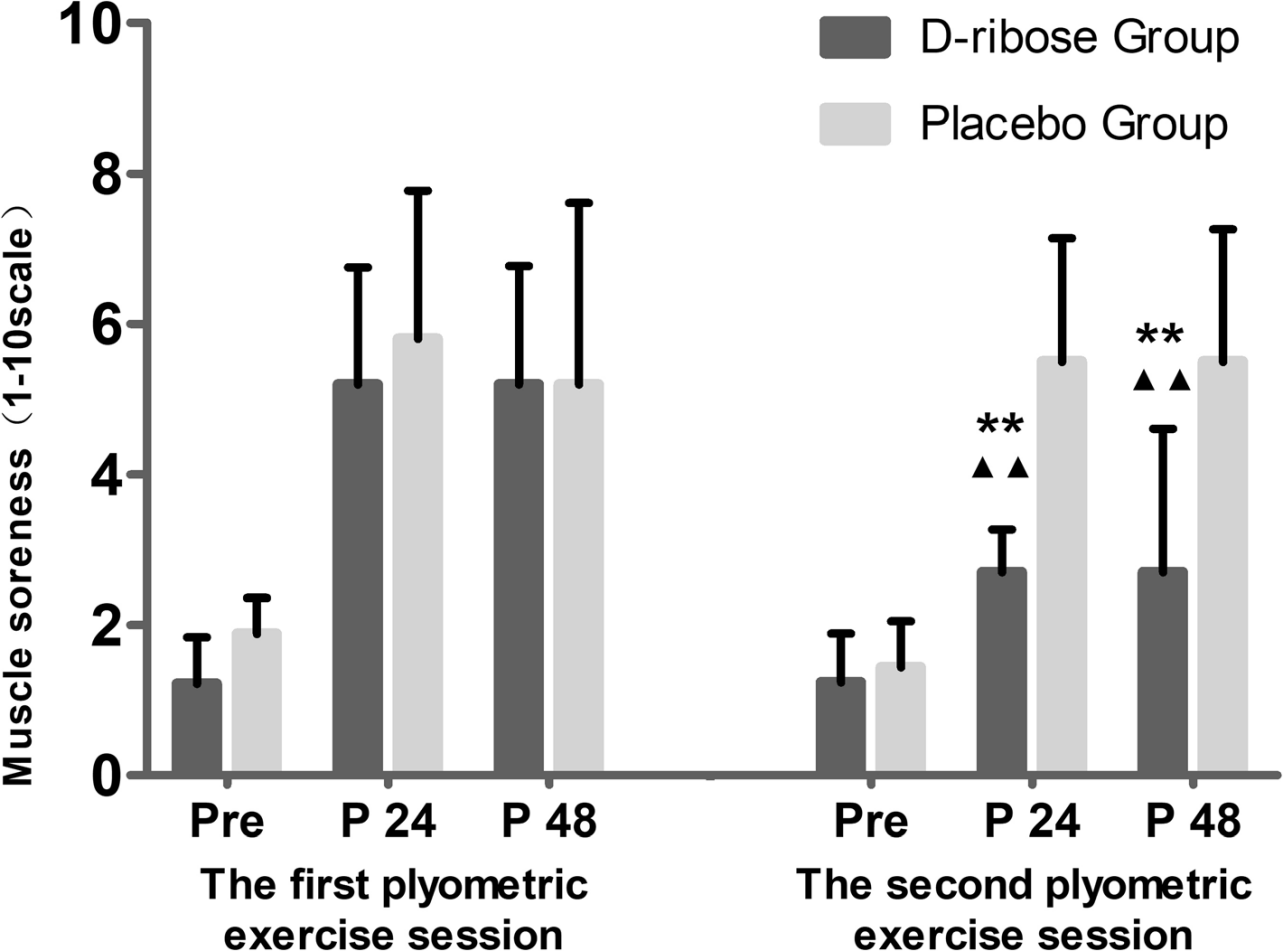
Muscle soreness in two plyometric exercise sessions. ** indicates a significant difference vs. Placebo group (*p* < 0.01). ^▲▲^indicates a significant difference vs. The first plyometric exercise session (*p* < 0.01)

At the second test, muscle soreness of DRIB was significantly lower than that of PLAC (*p* < 0.01), and was significantly lower after 24-h and 48-h in the second exercise session than in the first exercise session (*p* < 0.01) (Fig. 3).

### Isokinetic muscle strength

Changes in FTW, ETW, FPT and EPT cytokines are summarized in Table 2. Similar changes were found in both groups in FTW and FPT, with no significant difference either between (*p* > 0.05) or within groups (*p* > 0.05). ETW decreased significantly in PLAC after 24-h (*p* < 0.01) and 48-h (*p* < 0.01) but not in DRIB (*p* > 0.05). EPT decreased significantly in PLAC after 24-h (*p* < 0.01) and 48-h (*p* < 0.05), decreased in DRIB significantly after 24-h (*p* < 0.01), and returned to the pre-exercise level before exercise (*p* > 0.05). No significant differences were found between groups at any time-points (*p* > 0.05).

**Table 2:** Comparison of pre-exercise (mean ± SD) and mean (±95% CI) changes for isokinetic muscle strength markers

Measure	Pre	Change DRIB	Change PLAC	Group p-value
Flexor total work(J)	496.14(96.82)			
P 24		18.67(−72.34, 29.61)	−13.14(−37.36, 69.56)	0.515
P 48		8.95(− 59.53, 36.26)	− 18.74(− 28.53, 71.93)	0.557
Extensor total work(J)	747.19(162.77)			
P 24		− 73.46(−2.21, 153.12)	− 146.49(62.84, 225.76)^b^	0.281
P 48		− 58.1(−18.04, 138.23)	− 117.49(33.36, 197.24)^b^	0.372
Flexor peak torque(N·m)	104.48(15.90)			
P 24		3.71(−11.18, 5.36)	−4.78(− 4.77, 12.57)	0.323
P 48		3.16(−10.80, 6.07)	−5.68(− 4.05, 13.65)	0.303
Extensor peak torque(N·m)	188.95(38.33)			
P 24		−19.68(5.66, 39.06)^b^	−39.35(18.89, 53.91)^b^	0.211
P 48		−13.41(−2.73, 34.91)	−25.25(2.56, 42.04)^a^	0.477

*DRIB* D-ribose group, *PLAC* Placebo group

^a^95% CI changes that failed to cross 0 indicates a significant difference vs. Pre

^b^99% CI changes that failed to cross 0 indicates a significant difference vs. Pre

### Muscle damage

Changes in blood related muscle damage markers are summarized in Table 3. CK increased significantly in both groups after 24-h (*p* < 0.01) and at 48-h (*p* < 0.01); CK was significantly higher in PLAC than in DRIB after 24-h (*p* < 0.01) but not at 48-h (*p* > 0.05). LDH increased significantly in PLAC after 24-h (*p* < 0.01) and 48-h (*p* < 0.01) but not in DRIB (*p* > 0.05); LDH was significantly higher in PLAC than in DRIB after 24-h (*p* < 0.01) and 48-h (*p* < 0.01). MB in both groups increased significantly after 24-h (*p* < 0.01), and then decreased significantly after 48-h (*p* < 0.01), but was significantly higher at 48-h than before exercise (*p* < 0.01); MB was significantly higher in PLAC than in DRIB after 24-h (*p* < 0.01).

**Table 3:** Comparison of baseline (mean ± SD) and mean (±95% CI) changes for blood related markers

Measure	Pre	Change DRIB	Change PLAC	Group p-value
Creatine kinase(U·L^− 1^)	71.52(12.09)			
P 24		98.88(− 127.87, −71.90)^a^	153.74(− 181.98, − 123.28)^a^	0.002^c^
P 48		81.21(− 135.65, − 28.77)^a^	138.17(− 193.11, − 81.02)^a^	0.079
Lactate Dehydrogenase(U·L^− 1^)	142.18(50.74)			
P 24		49.17(−98.68, 5.17)	114.97 (− 172.08, − 63.16)^a^	0.005^c^
P 48		34.01(−73.42, 10.21)	162.59(− 209.09, − 121.38)^a^	< 0.001^c^
Myoglobin (ng·ml^− 1^)	46.55(26.85)			
P 24		50.66(− 113.81, − 55.37)^a^	86.44(− 54.14, − 15.61)^a^	0.006^c^
P 48		16.45(− 124.11, −62.82)^a^	29.85(−60.99, − 20.58)^a^	0.230
Superoxide dismutase(U·ml^− 1^)	48.37(7.57)			
P 24		11.91(−17.92, − 4.8)^a^	11.6(− 40.12, − 15.25)^a^	0.932
P 48		28.23(− 19.09, − 5.32)^a^	16.66(−30.3, − 4.22)^a^	0.077
Total antioxidant capacity(U·ml^− 1^)	3.59(2.19)			
P 24		21.48(−30.16, − 12.06)^a^	14.97(−23.49, − 10.48)^a^	0.157
P 48		17.36(−24.87, − 5.89)^a^	13.11(− 20.34, − 6.7)^a^	0.248
Malondialdehyde(U·ml^− 1^)	4.02(1.39)			
P 24		1.37(− 3.98, 0.2)	3.75(−1.33, 0.78)^a^	0.029^b^
P 48		−0.25(− 5.36, − 0.98)	2.87(− 3.41, − 1.19)^a^	< 0.001^c^

*DRIB* D-ribose group, *PLAC* Placebo group

^a^99% CI changes that failed to cross 0 indicates a significant difference vs. Pre

^b^indicates a significant difference vs. Placebo group (*p* < 0.05)

^c^indicates a significant difference vs. Placebo group (*p* < 0.01)

### Oxidative stress

Changes in blood related oxidative stress markers are summarized in Table 3. SOD increased in significantly in both groups after 24-h (*p* < 0.01), and remained significantly elevated in PLAC after 48-h (*p* < 0.01) but not in DRIB (*p* > 0.05). However, DRIB SOD was significantly higher than before exercise (*p* < 0.01). T-AOC increased in significantly in both groups after 24-h (*p* < 0.01), and remained significantly elevated in PLAC after 48-h (*p* < 0.01) but not in DRIB (*p* > 0.05). However, DRIB T-AOC was significantly higher than before exercise (*p* < 0.01). MDA increased significantly in PLAC after 24-h (*p* < 0.01) and 48-h (*p* < 0.01) but not in DRIB (*p* > 0.05). MDA was significantly higher in PLAC than in DRIB after 24-h (*p* < 0.05) and 48-h (*p* < 0.01).

## Discussion

The purpose of this study was to explore whether timed D-ribose supplementation could reduce DOMS-associated factors and help to speed DOMS recovery. Because a number of markers enable a comprehensive picture of muscle status, we assessed multiple markers to better understand muscle stress and to better discern the potential impact of D-ribose supplementation on DOMS [17]. We hypothesized that D-ribose supplementation would be effective in alleviating symptoms and markers of DOMS, as reflected by muscle performance and blood related markers. Our findings demonstrated that oral administration of 15 g D-ribose compared to a placebo at the 5 time-points 1-h before, 1-h, 6-h, 12-h and 36-h after exercise reduced lower limbs muscle soreness, improved the degree of knee extensor function, lowered muscle damage and the degree of lipid peroxidation, and accelerated the recovery of DOMS.

During the recovery period (24-h and 48-h after exercise) after the first exercise session, average muscle soreness of subjects in both groups was 5–6, suggesting that the plyometric exercise protocol in this study would induce DOMS. The muscle soreness in this study is slightly higher than that in earlier studies (∼3–4) [12, 39]. This is likely the result of the protocol requiring greater exercise frequency (140 versus 100) and shorter rest intervals (90s versus 120 s). During the recovery period (24-h and 48-h after exercise) after the second exercise session, we found that muscle soreness in PLAC was similar to that of the first exercise session, while DRIB was significantly lower than that of the first exercise session, and was also significantly lower than that of PLAC. These results suggest that D-ribose supplementation enabled the alleviation of DOMS-associated soreness during the post-exercise period.

The present study showed that ETW (0.82, 0.86) and EPT (0.81, 0.89) in PLAC decreased during DOMS (after 24-h and 48-h) and didn’t recover to the before-exercise level (ETW, *p* < 0.01; EPT, *p* < 0.05). This result corresponds to a previous study, which found that DOMS decreased peak torque and total work [40]. In contrast, DRIB group performed better, with no change in ETW (0.91, 0.92) and EPT (0.89, 0.92) within 48-h after exercise. This may reflect the effect of D-ribose stimulates the recovery process of knee extensor strength. In both DRIB and PLAC no change was shown in FTW and FPT of knee flexor (*p* > 0.05). As mentioned above, plyometric exercise includes eccentric contraction and concentric contraction, with the former is more prone to DOMS [3, 12]. Thus, after our exercise session, the decrease of knee flexor strength was not as severe as that of knee extensor. No difference was found in any maker within 48-h recovery period between groups, and we speculate that this may be related to the supplemental dose or to the muscle characteristics of subjects. We have not found any studies that measured the muscle performance of D-ribose supplementation post-exercise during DOMS, suggesting that further investigation could help to clarify this issue.

It has been established that skeletal muscle mechanical damage could lead to an increase of cell membrane permeability resulting in an increase in blood markers like CK and MB, which are sensitive markers of muscle damage [12, 17, 41–43]. In the present study, serum LDH, CK and MB in PLAC increased after exercise and did not return to the baseline data during the recovery time of 48-h after exercise. CK in our study increased to the peak value 24-h after exercise, which was earlier than the 48-h peak of CK response after exercise considered by previous studies [42]. One possibility is that our plyometric exercise may have caused greater muscle damage, resulting in a more rapid CK increase. LDH in PLAC increased continuously within the 48-h following exercise, which was consistent with earlier studies [12, 42], but was different than the later rise of CK and MB. CK and MB was significantly lower after exercise in DRIB compared to PLAC, reflecting that D-ribose supplementation accelerated the restoration of membrane integrity and reduced the leakage of enzyme from muscle to blood [24].

Addis et al. (2012) [20] had demonstrated that D-ribose supplementation enhanced the recovery of high-energy phosphate after stress and seemed to offer additional benefits by reducing the formation of free radicals. Our results support this point. As a product of lipid peroxidation, MDA was significantly lower in DRIB than in PLAC, indicating that ROS production was inhibited. In the present study, SOD (antioxidant enzyme) and T-AOC (antioxidant capacity) in DRIB showed no difference from PLAC, which may indicate that supplementation of D-ribose has a limited effect on improving antioxidant capacity. It is also possible that, since we did not record the dietary intake of the subjects during the study, they may have consumed ample antioxidants on their own, which could have blunted the potential antioxidant impact of D-ribose.

## Limitation

It is a limitation that the study was not a double-blind cross-over study. However, cross-over design is challenging studying eccentric exercise because adaptation occurs during the first sessions. Despite all this, as a single-blind study with a relatively small N and no capability of fully assessing subject adherence to the protocol, future studies should try to reduce these limitations through greater controls, a double-blind crossover protocol, and increased sample size.

It is also a limitation that the experiment did not include controlling the subjects’ daily diet or recording all food and fluid intake during the study period. The intake of natural food such as fruits or vegetables that contain nutrients with anti-inflammatory or antioxidant properties may also influence the results [44]. Therefore, it is recommended that future studies should perform a full assessment of subject diets should be included to better understand the results and more accurately validate the differences between groups.

## Conclusion

D-ribose supplementation may safely help to alleviate DOMS induced by plyometric exercise in the general population. The supplementation with D-ribose reduces muscle soreness, enhances recovery of muscle damage, and inhibit the formation of lipid peroxides.

## Data Availability

The datasets generated and/or analyzed as part of the current study are not publicly available due to confidentiality agreements with subjects. However, they can be made available solely for the purpose of review and not for the purpose of publication from the corresponding author upon reasonable request.

## Abbreviations

DOMS: Delayed onset muscle soreness
DRIB: D-ribose group
PLAC: Placebo group
ROS: Reactive oxygen species
ATP: Adenosine triphosphate
PPP: Pentose phosphate pathway
CK: Creatine kinase
LDH: Lactate dehydrogenase
MB: Myoglobin
SOD: Superoxide dismutase
T-AOC: Total antioxidant capacity
MDA: Malondialdehyde
FPT: Flexor peak torque
EPT: Extensor peak torque
FTW: Flexor total work
ETW: Extensor total work
